# Relationship between initiation time of adjuvant chemotherapy and survival in ovarian cancer patients: a dose-response meta-analysis of cohort studies

**DOI:** 10.1038/s41598-017-10197-1

**Published:** 2017-08-25

**Authors:** Yi Liu, Tiening Zhang, Qijun Wu, Yisheng Jiao, Tingting Gong, Xiaoxin Ma, Da Li

**Affiliations:** 1 Department of Obstetrics and Gynecology, Shengjing Hospital of China Medical University, Shenyang, China; 2 Department of Pediatrics, Shengjing Hospital of China Medical University, Shenyang, China; 3 Department of Clinical Epidemiology, Shengjing Hospital of China Medical University, Shenyang, China

## Abstract

Although several studies have previously investigated the association between the initiation time of adjuvant chemotherapy and survival in ovarian cancer, inconsistencies remain about the issue. We searched PubMed and Web of Science through the May 24, 2017 to identify cohort studies that investigated the aforementioned topic. Fourteen studies with 59,569 ovarian cancer patients were included in this meta-analysis. We conducted meta-analyses comparing the longest and shortest initiation time of adjuvant chemotherapy and dose-response analyses to estimate summary hazards ratios (HRs) and 95% confidence intervals (CIs). A random-effects model was used to estimate HRs with 95% CIs. When comparing the longest with the shortest category of initiation time of adjuvant chemotherapy, the summary HR was 1.18 (95% CI: 1.06–1.32; *I* ^2^ = 17.6; *n* = 7) for overall survival. Additionally, significant dose-response association for overall survival was observed for each week delay (HR = 1.04; 95% CI: 1.00–1.09; *I* ^2^ = 9.05; *n* = 5). Notably, these findings were robust in prospective designed cohort studies as well as studies with advanced stage (FIGO III-IV) patients. No evidence of publication bias was observed. In conclusion, prolonged initiation time of adjuvant chemotherapy is associated with a decreased overall survival rate of ovarian cancer, especially in patients with advanced stage ovarian cancer.

## Introduction

Epithelial ovarian cancer is the most lethal gynecologic malignancy^1, 2^, with nearly 22,280 new cases diagnosed in the United States resulting in 14,240 deaths in 2016^2^. It is the seventh leading cause of cancer and the eighth leading cause of cancer related deaths among women worldwide^1^. Approximately 75% of patients with this disease are diagnosed in advanced stages^3^ which were probably attributed to no specific clinical manifestations and effective screening methods^4^. Relative survival at 5 years was 89%, 70%, 36%, and 17%, and at 10 years relative survival was 84%, 59%, 23%, and 8% for stages I, II III, and IV, respectively^5^. Due to the relatively poor prognosis and lack of standard treatment for advanced ovarian cancer currently, primary tumor resection surgery followed by Platinum-taxane chemotherapy is the most common and accepted treatment method^6^.

Several experimental studies^7–9^ showed that the remove of the primary cancer could promote cancer growth; likewise, time interval from surgery to chemotherapy influence the growth of metastasis, and an earlier start of chemotherapy offered a remarkable advantage in preventing systemic relapse. However, the relationship between initiation time of adjuvant chemotherapy in ovarian cancer and prognosis from epidemiological studies has remained controversial. Some studies^10–12^ suggested that shorter initiation time of adjuvant chemotherapy seems to have a predictive value for the prognosis of ovarian cancer patients. Nevertheless, other studies^13–15^ did not draw a clear conclusion about the aforementioned association. Additionally, there might be a difference in results when stratified by residual disease^16, 17^ which was one of the most important prognostic factors of ovarian cancer. A recent systematic literature review performed by Alexander *et al*.^18^ showed that the optimal initiation time of adjuvant chemotherapy in ovarian cancer is unclear. However, without quantitative findings and subgroup and sensitivity analyses might limit the interpretation of their results. To the best of our knowledge, there has been no quantitative assessment and dose-response analysis of published findings on this topic. Notably, several important studies^13, 19, 20^ with large sample sizes were published after their study. Herein, to update the evidence as well as further clarify the relationship between initiation time of adjuvant chemotherapy and the prognosis of ovarian cancer patients, we carried out this dose-response meta-analysis of epidemiological studies.

## Result

### Search results and characteristics of included studies

**Figure 1:**
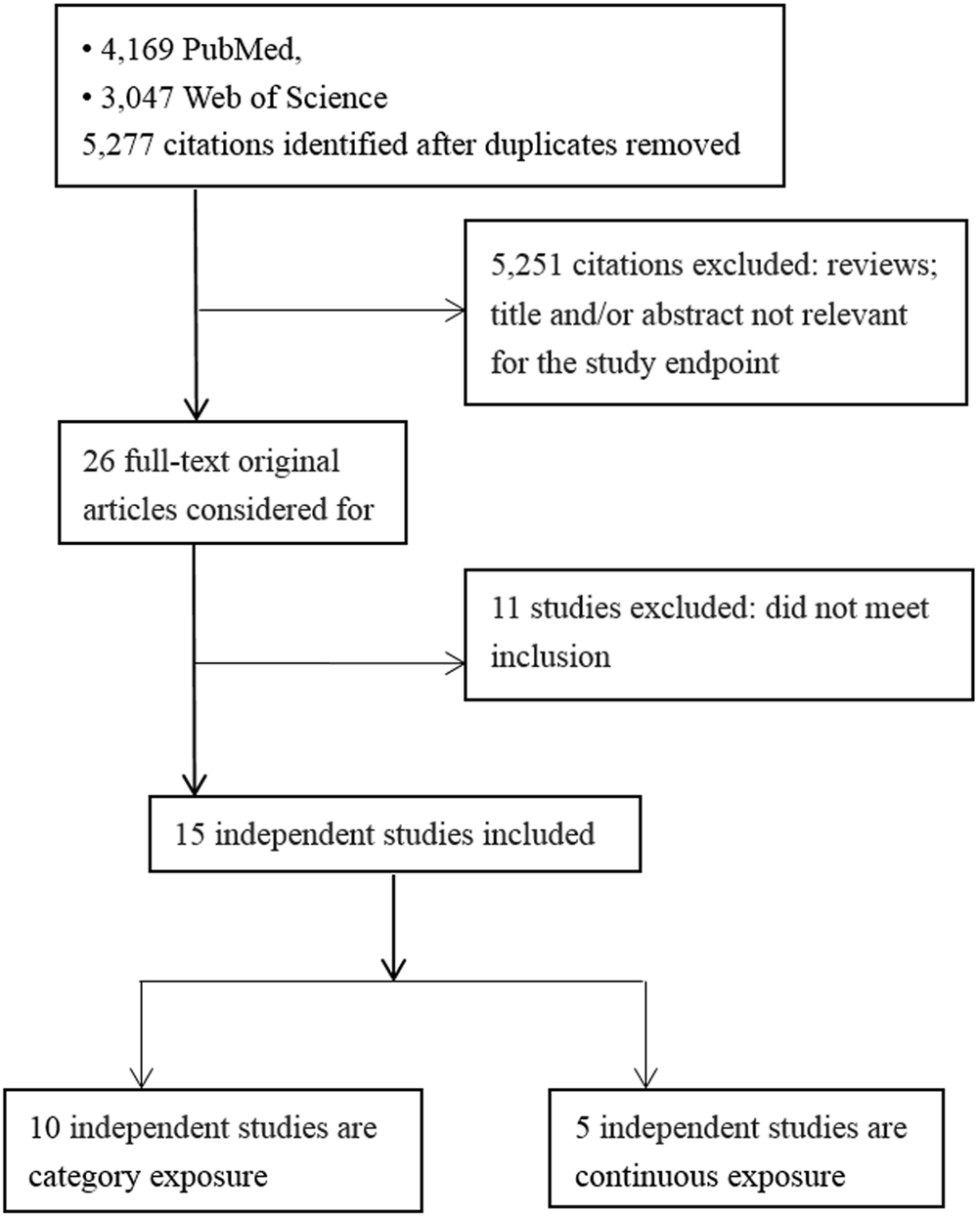
Flow-chart of study selection.

The literature search strategy yielded 5,277 studies for eligibility from the databases of PubMed and Web of Science. After carefully screening each title and abstract, 26 studies were included for full reviews of original articles. Of these, 11 were excluded because of the inclusion and exclusion criteria. Finally, 15 studies^10–13, 15, 19–28^ were included in the present meta-analysis (Fig. 1). The detail characteristics of these 15 studies are demonstrated in the Table S1 in the supplement. A total of 59,569 ovarian cancer cases were included.

### Quality assessment

The methodological quality of all studies is depicted in the Table S2 in the supplement. The major differences among these studies were comparability and outcome, especially in adjustments for potential confounders and duration of follow-up. Only five of 15 studies adjusted for two defined confounders (FIGO stage and residual disease) and received full scores. Additionally, ten studies followed their ovarian cancer patients more than twenty-four months.

### Longest compared with shortest category of initiation time of adjuvant chemotherapy

**Figure 2:**
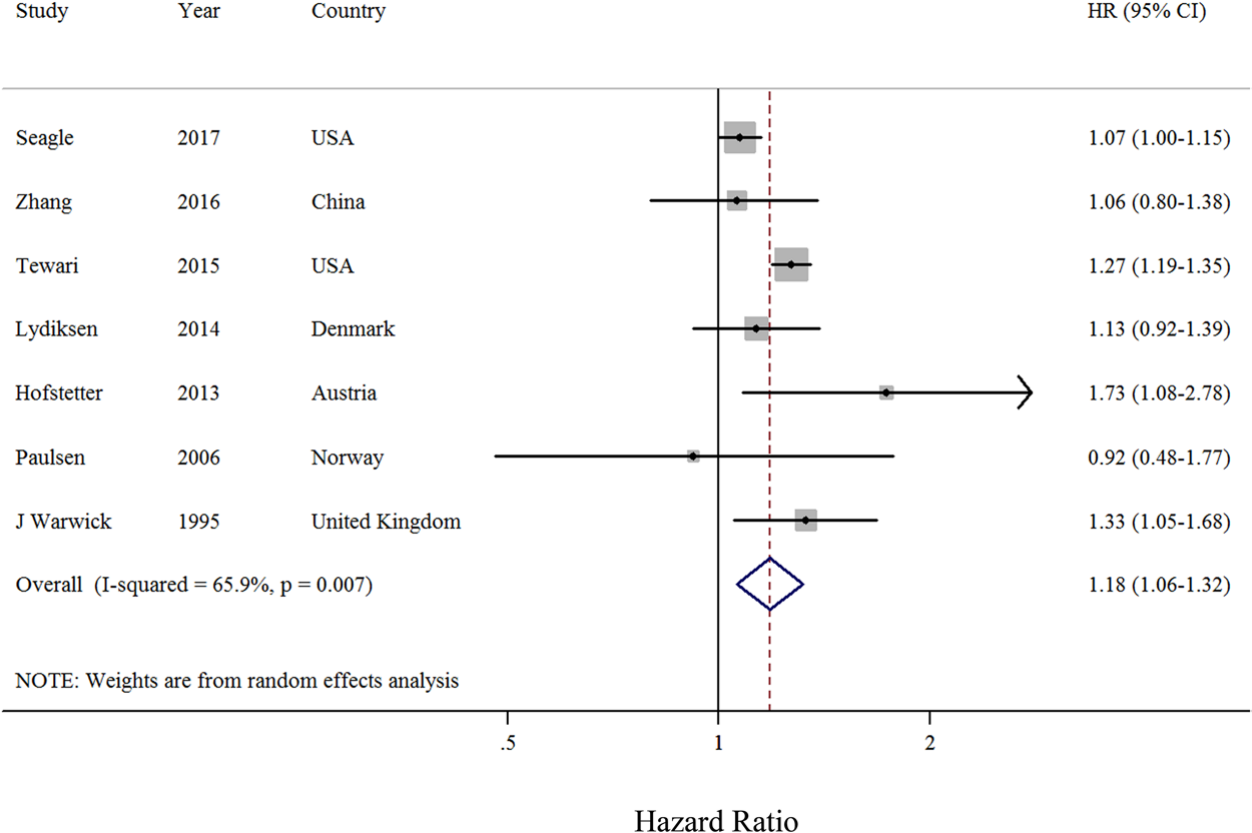
Forest plots of the relationship between the interval from surgery to chemotherapy and overall survival of patients with ovarian cancer (longest compared with shortest interval). Squares indicate study-specific risk estimates (size of the square reflects the study-specific statistical weight); horizontal lines indicate 95% CIs; diamonds indicate the summary hazards ratio with its 95% CI. HR: hazards ratio.

Six observational studies provided the data on the longest compared with the shortest initiation time of adjuvant chemotherapy. The summary HR of OS was 1.16 (95% CI: 1.04–1.29) with moderate heterogeneity (*I* ^2^ = 67%, *P* = 21.2) (Fig. 2). There was no evidence of publication bias, both quantitatively (*P* = 0.88 for Begg and *P* = 0.16 for Egger) and qualitatively, on visual inspection of the funnel plot.

Several post hoc subgroup analyses stratified by study characteristics and adjustment for potential confounders were carried out (Table 1). In five prospective studies, longer initiation time of adjuvant chemotherapy was associated with a significant 22% risk decrease in OS of ovarian cancer. Subgroup analysis on geographic location, number of cases, FIGO stage, and chemotherapy, yielded similar results in European studies, studies with more than 600 cases, studies with FIGO III–IV stage patients, and studies with platinum based plus Taxane chemotherapy. Additionally, when stratified by whether there had been an adjustment for potential confounders, similar results were observed in studies with adjustment for FIGO and residual disease. Notably, there was no evidence of significant heterogeneity between subgroup analyses in meta-regression analysis. In a sensitivity analysis, we sequentially removed one study at a time and reanalyzed the data. The seven study-specific HRs ranged from a low of 1.14 (95% CI: 1.02–1.27, *I* ^2^ = 7.05%, *P* = 0.22) after omitting the study by Seagle *et al*.^25^ to a high of 1.25 (95% CI: 1.16–1.34, *I* ^2^ = 5.48%, *P* = 0.36) after omitting the study by Feng *et al*.^13^.

**Table 1:** Summary risk estimates of the associations between initiation time of adjuvant chemotherapy and overall survival in ovarian cancer patients (longest versus shortest).

	No. of Study	Summary HR	95% CI	*I* ^2^ (%)	*P*_h_^†^	*P*_h_^‡^
**Overall survival**	8	1.16	1.04–1.29	67	21.2	
**Subgroup analyses**						
**Study design**						0.02
Prospective	5	1.22	1.02–1.46	48.7	0.10	
Retrospective	3	1.08	1.01–1.15	0	0.88	
**Geographic location**						0.45
Asia	1	1.06	0.80–1.38	N/A	N/A	
North America	3	1.11	0.94–1.32	87.7	0	
Europe	4	1.25	1.05–1.49	21.6	0.28	
**Number of cases**						0.77
<600	4	1.16	0.83–1.62	60.6	0.06	
≥600	4	1.15	1.02–1.29	77.6	0	
**FIGO stage**						0.93
All	3	1.09	0.93–1.28	0	0.81	
III–IV	4	1.23	1.07–1.42	81.5	0	
I–II	1	0.78	0.51–1.19	N/A	N/A	
**Residual disease**						
Yes	1	0.69	0.30–1.60	N/A	N/A	
No	1	1.35	0.51–3.57	N/A	N/A	
**Chemotherapy**						0.66
Platinum based	2	1.26	0.98–1.63	7.8	0.30	
Platinum based plus Taxane	5	1.17	1.04–1.32	65.9	0.01	
**Adjustment for potential confounders**						
**FIGO**						0.58
Yes	7	1.16	1.04–1.30	70.9	0.26	
No	1	0.92	0.48–1.77	N/A	N/A	
**Histology**						0.58
Yes	5	1.07	0.91–1.26	36.7	0.18	
No	3	1.26	1.19–1.34	0	0.51	
**Residual Disease**						0.37
Yes	5	1.22	1.10–1.36	23.5	0.26	
No	3	1.09	0.89–1.34	62.6	0.07	

Abbreviations: CI, confidence interval; FIGO, International Federation of Gynecology and Obstetrics; HR, hazards ratio; N/A, not available.

### Dose-response analysis of initiation time of adjuvant chemotherapy

**Figure 3:**
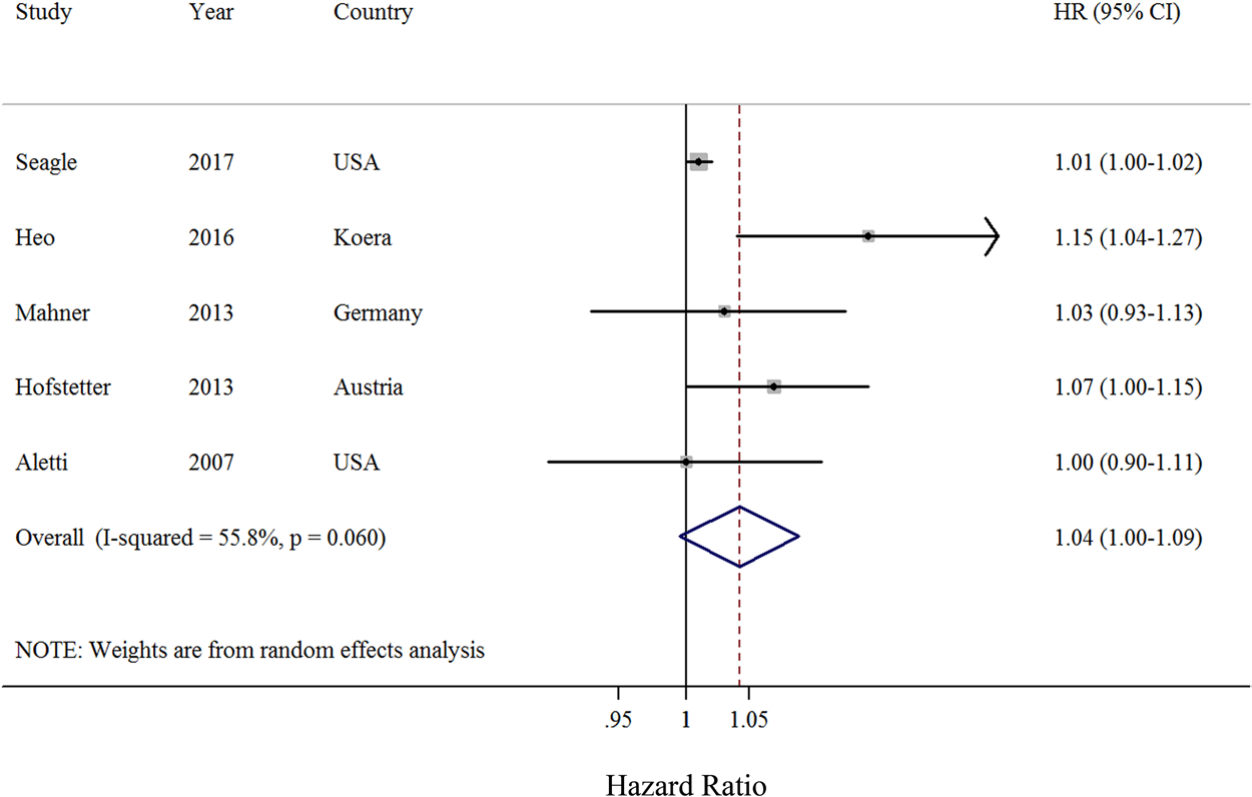
Forest plots of the relationship between the interval from surgery to chemotherapy and overall survival of patients with ovarian cancer (per week increments). Squares indicate study-specific risk estimates (size of the square reflects the study-specific statistical weight); horizontal lines indicate 95% CIs; diamond indicates the summary hazards ratio with its 95% CI. HR: hazards ratio.

Four observational studies provided the sufficient data on dose-response analysis of initiation time of adjuvant chemotherapy. The summary HR was 1.04 (95% CI: 1.00–1.09) with moderate heterogeneity (*I* ^2^ = 55.8%, *P* = 0.06) (Fig. 3). There was no evidence of publication bias, both quantitatively (*P* = 0.62 for Begg and *P* = 0.65 for Egger) and qualitatively, on visual inspection of the funnel plot.

Although positive associations were observed in the majority of these subgroup analyses, not all of them showed statistical significance (Table 2). Similar to the results of the longest versus the shortest analysis, there was no evidence of significant heterogeneity between subgroup analyses in meta-regression analysis. In a sensitivity analysis, we sequentially removed one study at a time and reanalyzed the data. The 4 study-specific HRs ranged from a low of 1.01 (95% CI: 1.00–1.02, *I* ^2^ = 2.75%, *P* = 0.43) after omitting the study by Heo *et al*.^19^ to a high of 1.06 (95% CI: 1.01–1.12, *I* ^*2*^ = 4.12%, *P* = 0.25) after omitting the study by Seagle *et al*.^25^.

**Table 2:** Summary risk estimates of the associations between initiation time of adjuvant chemotherapy and overall survival in ovarian cancer patients (per week increments).

	No. of Study	Summary HR	95% CI	*I* ^2^ (%)	*P*_h_^†^	*P*_h_^‡^
**Overall survival**	5	1.04	1.00–1.09	55.8	0.06	
**Subgroup analyses**						
**Study design**						0.82
Prospective	2	1.06	1.00–1.12	0	0.53	
Retrospective	3	1.04	0.97–1.13	69.1	0.04	
**Geographic location**						0.29
Asia	1	1.15	1.04–1.27	N/A	N/A	
North America	2	1.01	1.00–1.02	0	0.86	
Europe	2	1.06	1.00–1.12	0	0.53	
**Number of cases**						0.13
<600	3	1.07	1.00–1.15	44.2	0.17	
≥600	2	1.01	1.00–1.02	55.8	0.07	
**FIGO**						0.84
All	1	1.03	0.93–1.14	N/A	N/A	
III-IV	4	1.05	0.99–1.11	66.4	0.03	
**Residual disease**						
Yes	1	1.09	1.01–1.78	N/A	N/A	
No	1	0.98	0.94–1.03	N/A	N/A	
**Chemotherapy**						0.19
Platinum based	1	1.00	0.90–1.11	N/A	N/A	
Platinum based plus Taxane	3	1.02	0.99–1.05	26.2	0.26	
N/A	1	1.15	1.04–1.27	N/A	N/A	
**Adjustment for potential confounders**						
**FIGO**						0.84
Yes	4	1.05	0.99–1.11	66.4	0.03	
No	1	1.03	0.93–1.14	N/A	N/A	
**Histology**						0.48
Yes	3	1.02	0.99–1.05	26.2	0.26	
No	2	1.07	0.94–1.23	72.0	0.06	
**Residual Disease**						0.65
Yes	1	1.07	1.00–1.15	N/A	N/A	
No	4	1.04	0.98–1.09	54.6	0.09	

Abbreviations: CI, confidence interval; FIGO, International Federation of Gynecology and Obstetrics; HR, hazards ratio; N/A, not available.

## Discussion

The present meta-analysis comprehensively and systematically summarized the evidence from 15 cohort studies and found that early initiation of chemotherapy will improve the OS rates of patients with ovarian cancer. Relative OS decreases by 4% for each week in delay of initiating adjuvant chemotherapy. This finding was consistent in patients with FIGO III-IV stage. In the clinical practice, initiation time of adjuvant chemotherapy may need to be carefully considered by physicians when discussing adjuvant chemotherapy with ovarian cancer patients after surgery.

Currently, primary tumor resection surgery followed by platinum-taxane chemotherapy has been the most common and well-accepted treatment method for ovarian cancer patients^6^ while the optional initiation time of adjuvant chemotherapy has remained unclear. A recent systematic literature review carried out by Alexander *et al*.^18^ evaluated the association between initiation time of adjuvant chemotherapy and survival in six priority cancers. However, limited evidence of ovarian cancer was provided which might be attributed to a lack of quantitative assessments, dose-responses, subgroups and sensitivity analyses in their study. Additionally, since the relatively earlier search date of their study (April 2014), in the recent two years several studies with large sample sizes have been published. For example, Tewari *et al*.^20^ carried out a post-trial ad hoc analysis on the basis of 1,718 ovarian cancer patients from a phase III randomized, double-blind, placebo-controlled trial. They suggested that initiation time of adjuvant chemotherapy was predictive of overall survival. Notably, when initiation time of adjuvant chemotherapy exceeded 25 days, the complete resection group (i.e., stage IV) encountered an increased risk of death. Additionally, two recent retrospective cohort studies^13, 19^ from Asia published their results, although one of them is a meeting abstract^19^. Several potential biological theoretical rationales may explain the benefits of initiating adjuvant chemotherapy without delay after curative surgery. Gunduz *et al*.^9^ suggested that resection of the tumor could increase the tumor growth which may result from a conversion of non-cycling cells in G_0_ phase into proliferation. Similarly, surgery has been shown to temporarily modulate the function of the immune system; for instance, antitumor effects of interleukin-2 and lymphokine-activated killer cells will be significantly reduced by the performance of a laparotomy^29^. Additionally, in animal models, peri-operative chemotherapy demonstrated a significant advantage in preventing systemic relapse and a short time span to chemotherapy resulted in the most effective control of metastases^7, 8^. After primary surgery, a large proportion of tumor cells will grow rapidly, and thus, the earlier chemotherapy is started, the greater the chance that the chemotherapy treatment will control the residual disease.

To the best of our knowledge, this is the first meta-analysis comprehensively and quantitatively evaluating the effect of initiation time of adjuvant chemotherapy in ovarian cancer patients. On the basis of 59,569 cases from 15 cohort studies, we have sufficient statistical evidence to detect the aforementioned association and further reinforce earlier results of previous meta-analysis. Additionally, numerous subgroup analyses were carried out to increase the robustness as well as to explore the heterogeneity. Notably, we first provided evidence that advanced ovarian cancer patients (FIGO III–IV) should consider shorter initiation time of adjuvant chemotherapy to improve their survival. Our meta-analysis also had several limitations. Firstly, we included both prospective and retrospective cohort studies in the present meta-analysis. However, compared to prospective cohort studies, retrospective cohort studies might be limited by recall bias. Although we found no evidence of differences between these two study designs, the point estimate of prospective cohort studies was slightly stronger than retrospective cohort studies (1.22 versus 1.08). Secondly, administration of chemotherapy could be delayed by various factors including purpose of patients, age, performance status, and peri-operative co-morbidity in clinical practice that might impact the outcomes. Although positive findings were observed in subgroup analyses stratified by whether there was an adjustment for potential confounders (FIGO, histology, and residual disease), not all of them showed significance which might be partly attributed to the limited included studies. Therefore, we could not rule out the possibility that there might exist other potential confounders that not be adjusted by most of the studies affecting the results. Thus, further analyses adjusting or stratification for more confounders are needed. Thirdly, since there are no specific clinical manifestations and effective screening methods, over 75% ovarian cancer patients are diagnosed in advanced stages^3^. For example, although six studies mentioned that they included patients in all stages of the disease, the majority of these cases were in the advanced stages of the disease. The findings of the present meta-analysis should be interpreted with caution in patients with early stage ovarian cancer. Additionally, small-study bias, such as publication bias, might be an issue in meta-analyses, however, we failed to detect statistical evidence in the present study. Finally, although dose-response analyses were carried out in the present study, considering the limited available data, we failed to evaluate whether there was evidence for a nonlinear aforementioned association.

In conclusion, the present meta-analysis suggests that early initiation of adjuvant chemotherapy after surgery will improve the overall survival rates of ovarian cancer patients, especially in patients with advanced stage cancer. Future prospective clinical trials randomizing patients to different time intervals are warranted to confirm our findings as well as to further clarify the definitive relevance of the time between surgery and chemotherapy. Consequently, longer initiation time of adjuvant chemotherapy should be advised as little as possible in clinical practice when treating ovarian cancer patients after surgery, especially for these patients with advanced stage cancer.

## Materials and Methods

### Search strategy

Two investigators (YiLiu and Tiening Zhang) carried out a systematic literature search of PubMed and Web of Science databases and extracted data up to May 24, 2017 for all relevant epidemiological studies without restriction. The language restriction was imposed on English and following keywords were employed in the search: (ovary OR ovarian) AND (cancer OR neoplasm OR tumor OR carcinoma) AND (time OR interval) AND (survival OR mortality). We followed standard criteria for conducting and reporting this meta-analysis^30^. Additionally, we also included an unpublished study^19^ and searched the reference sections of the studies that were included in our study to identify publications for further potentially relevant articles^31–34^.

### Study selection criteria

Titles, abstracts, and articles for eligibility using the following criteria was reviewed by two investigators (YiLiu and Tiening Zhang) independently. The study was included if it accorded with following criteria (1) evaluated and clearly defined the time interval between primary surgery to inception of adjuvant chemotherapy as exposure, (2) reported ovarian cancer survival (progression-free or OS), (3) reported hazards ratios (HR) and 95% confidence interval (CIs) for aforementioned association. Studies were excluded if they: (1) were reviews without original data, ecological studies, editorials, or case reports and (2) failed to evaluate the aforementioned association as well as to report HR and 95% CI or the data necessary for calculating these risk estimates. Only data from the most recent comprehensive report were included when there were multiple publications from the same population. Any discrepancy was resolved by discussion with third investigator (Qijun Wu)^34–37^.

### Data extraction and quality assessmenth

From each study we extracted data on the first author, publication year, country, study design, numbers of cases within the cohorts, regimens of chemotherapy, tumor characteristics, time to chemotherapy (category/continuous), risk estimates and 95% CI and adjustment confounders. Extracted data were entered into a standardized Excel (Microsoft Corporation) file. We also sought supplementary appendixes of included studies^38–40^. Discrepancies were resolved by discussion. Data extraction was performed by YiLiu and confirmed independently by the other authors (Tiening Zhang and Qijun Wu) for accuracy.

Additionally, the methodological quality of included epidemiological studies was independently assessed by YiLiu using the Newcastle-Ottawa scale (NOS) and confirmed by the other investigator (Tiening Zhang)^41, 42^. According to the NOS, The included studies were evaluated across three categories: selection, comparability, and exposure/outcome. Since quality scoring of these included studies might not only hide important information by combining disparate study features into a single score, but also introduce an arbitrary subjective element into the analysis, we described these studies instead of dividing them into categories of high or low quality utilizing a scoring system.

### Statistical analysis

For studies that reported the risk estimates stratified by residual disease and race, we used a previously described counting method proposed by Hamling *et al*.^43^ to recalculate the HR and 95% CI. We used the main cut-off values mentioned in the manuscripts for studies that reported the risk estimates using different cut-off values. In dose-response analysis, for studies that reported the risk estimates for each day increment in initiation time of adjuvant chemotherapy, we used the methods first carried out by Danesh *et al*.^44^ to transfer the information into per week increments.

Since these included studies differed both clinically and methodologically, we report the results from the DerSimonian and Laird random effects models over the fixed effects model^45^. If no heterogeneity exits in the pooled data, results of random and fixed effects models are the same, and if significant heterogeneity is present, a random effects model is more conservative^45^. Therefore, the random effects model, which considered both within- and between-study variation, was used to calculate summarized HR and 95% CIs. I^2^ statistic was used to quantify the heterogeneity across studies. I^2^ > 50% indicated significant heterogeneity. Subsequently, to explore the heterogeneity as well as check the influence of various factors on initiation time of adjuvant chemotherapy of ovarian cancer survival, we further carried out post hoc subgroup analyses stratified by study design (prospective versus retrospective), geographical location (Asia, North America, and Europe), number of cases (<600 versus ≥ 600), FIGO (International Federation of Gynecology and Obstetrics) stage (all, III-IV versus I-II), residual disease (yes versus no), chemotherapy (Platinum based, Platinum based plus Taxane versus N/A), and adjustment for potential confounders (FIGO, histology, and residual disease). Heterogeneity between subgroups was evaluated by meta-regression, which can be used to explore reasons for heterogeneity when trials, not patients, are pooled^46^. Sensitivity analyses were carried out to examine the influence of individual data sets on the overall estimate by deleting each study in turn. Small-study bias, such as publication bias, was assessed with visually inspecting a funnel plot for asymmetry as well as by the test developed by Egger *et al*.^47^ and Begg and Mazumdar^48^. To avoid the results were driven by one large study or by a study with an extreme result, we carried out sensitivity analyses excluding one study at a time to explore whether All statistical analyses were performed with Stata 12.0 (StataCorp LP).

## Electronic supplementary material

Supplementary Information
