# Supplementary material for: Relationship between initiation time of adjuvant chemotherapy and survival in ovarian cancer patients: a dose-response meta-analysis of cohort studies
Source: Sci Rep. 2017 Aug 25;7:9461. doi: 10.1038/s41598-017-10197-1 (PMC5572704; doi:10.1038/s41598-017-10197-1)
Supplement: Supplementary file 1 — Supplementary Information [file 41598_2017_10197_MOESM1_ESM.doc]

**Relationship between initiation time of adjuvant chemotherapy and survival in ovarian cancer patients: a dose-response meta-analysis of cohort studies**

Yi Liu, Tie-Ning Zhang, Qi-Jun Wu, Yi-Sheng Jiao, Ting-Ting Gong, Xiao-Xin Ma, Da Li

**Supplementary Table S1. Characteristics of cohort studies included in the meta-analysis**

| **First Author, (Ref), Year, Country** | **Study Design** | **No. of Case** | **Chemotherapy** | **Tumor characteristics** | **Exposure categories** | **Risk Estimates**  **(95% CI)** | **Adjusted factors** |
| --- | --- | --- | --- | --- | --- | --- | --- |
| Seagle et al.^25^,2017, USA | Retrospective | 45001 | Platinum-based plus Taxane | All | OS (days)  ≥50 vs. <50 | 1.07 (1.00-1.15) | Age, comorbidity scores, distance to hospital, stage, grade, histology, race, cancer center type, insurance status, and income quartile |
|  |  |  |  |  |  |  |  |
| Chan et al.^26^,2016, USA | Prospective | 497 | Platinum-based  Platinum-based plus Taxane | I-II high risk | OS (weeks)  ≥4 vs. <4  PFS (weeks)  ≥4 vs. <4 | 0.78 (0.51-1.19)  0.72 (0.46-1.13) | Age, performance status, stage of disease, tumor  grade, cytology, and type of treatment |
|  |  |  |  |  |  |  |  |
| Garcia-Soto et al.^27^,2016, USA | Retrospective | 420 | Platinum-based plus Taxane | III | PFS (days) | 0.97 (0.94-1.00) | N/A |
|  |  |  |  |  |  |  |  |
| Feng et al.^13^, 2016, China | Retrospective | 625 | Platinum-based plus Taxane | High grade serous | OS (days)  ≥15 vs. <15  PFS (days)  ≥15 vs. <15 | 1.06 (0.80-1.38)  1.06 (0.87-1.29) | Age, FIGO stage, RD |
|  |  |  |  |  |  |  |  |
| Tewari et al.^20^, 2016, USA | Prospective | 1,718 | Platinum-based plus Taxane | FIGO III-IV | OS (days)  RD=micro  ≥40 vs. <40  RD ≤1cm  ≥40 vs. <40  RD >1cm  ≥40 vs. <40 | 1.30 (1.19-1.41)  1.17 (1.01-1.35)  1.24 (1.07-1.44) |  |
|  |  |  |  |  |  |  |  |
| Heo et al.^19^, 2015, Korea | Retrospective | 507 | N/A | FIGO III-IV | OS (per day) | 1.02 (1.00-1.03) | History of consultation to the department of general surgery, platinum resistance |
|  |  |  |  |  |  |  |  |
| Lydiksen et al.^28^, 2014, Denmark | Retrospective | 650 | Platinum-based plus Taxane | All | OS (days)  >32 vs. ≤32 | 1.13 (0.92-1.39) | RD and FIGO stage |
|  |  |  |  |  |  |  |  |
| Hofstetter et al.^10^, 2013, Austria | Prospective | 191 | Platinum-based plus Taxane | FIGO III-IV serous | OS (days)  ≤28 vs. >28  OS (per day) | 1.73 (1.08-2.78)  1.01 (1.00-1.02) | FIGO stage, postoperative RD, age, extent of surgery, and center |
|  |  |  |  |  |  |  |  |
| Mahner et al.^11^, 2013, Germany | Prospective | 3,326 | Platinum-based plus Taxane | All | RD=0  PFS (per week)  OS (per week)  RD=1  PFS (per week)  OS (per week) | 1.04 (0.97-1.11)  1.09 (1.01-1.78)  0.93 (0.90-0.97)  0.98 (0.94-1.03) | Study group, therapy arm and histologic subtype |
|  |  |  |  |  |  |  |  |
| Wright et al.^12^, 2012, USA | Retrospective | 3,991 | N/A | FIGO III-IV | CSS (weeks)  ≥12 vs. <12 | 1.32 (1.07-1.64) | Patient, tumor characteristics, perioperative complications |
|  |  |  |  |  |  |  |  |
| Aletti et al.^21^, 2007, USA | Retrospective | 218 | Platinum-based | FIGO IIIc-IV | OS (per day) | 1.00 (0.98-1.10) | N/A |
|  |  |  |  |  |  |  |  |
| Paulsen et al.^22^, 2006, Norway | Prospective | 371 | Platinum-based | All | OS (weeks)  RD=0  <6 vs. ≥6  RD=1  <6 vs. ≥6 | 1.35 (0.51-3.56)  0.69 (0.30-1.60) | Age, histology stage, ascites, hospital level, serious co-morbidity, cut of points of 2 or 4 weeks |
|  |  |  |  |  |  |  |  |
| Sorbe et al^23^, 2004, Sweden | Retrospective | 1,220 | Platinum-based | All | CSS (per day) | 1.00 (0.99-1.01) | FIGO stage, histology, tumor grade, RD |
|  |  |  |  |  |  |  |  |
| Flynn et al.^15^, 2002, United Kingdom | Prospective | 472 | Platinum-based | All | PFS (days)  <22 vs. ≥22 | 0.99 (0.79-1.24) | FIGO stage, bulk of RD, performance status |
|  |  |  |  |  |  |  |  |
| Warwick et al.^24^, 1995, United Kingdom | Prospective | 362 | Platinum-based | FIGO III-IV | OS (days)  ≤21 vs. >21 | 1.33 (1.05-1.68) | N/A |

Abbreviations: CI, confidence interval; CSS, cancer-specific survival; FIGO, International Federation of Gynecology and Obstetrics; N/A, not available; OS, overall survival; PFS, progression free survival; RD, residual disease.

**Supplementary Table S2. Methodological quality of cohort studies included in the meta-analysis**

| **First author (ref), publication year** | **Representativeness**  **of the exposed cohort** | **Selection of the unexposed cohort** | **Ascertainment of exposure** | **Outcome of interest not present at start of study** | **Control for important factor or additional factor ^a^** | **Assessment of outcome** | **Follow-up long enough for outcomes to occur ^b^** | **Adequacy of follow-up of cohorts ^c^** |
| --- | --- | --- | --- | --- | --- | --- | --- | --- |
| Seagle et al.^25^,2017 | * | * | * | * | * | * | * | * |
| Chan et al.^26^,2016 | * | * | * | * | * | * | - | * |
| Garcia-Soto et al.^27^,2016 | * | * | * | - | - | * | * | * |
| Feng et al.^13^, 2016 | * | * | * | * | ** | * | * | * |
| Tewari et al.^20^, 2016 | * | * | * | * | - | * | - | * |
| Heo et al.^19^, 2015 | * | * | * | * | - | * | - | * |
| Lydiksen et al.^28^, 2014 | * | * | * | * | ** | * | * | * |
| Hofstetter et al.^10^, 2013 | * | * | * | * | ** | * | * | * |
| Mahner et al.^11^, 2013 | * | * | * | * | * | * | * | * |
| Wright et al.^12^, 2012 | * | * | * | * | * | * | - | * |
| Aletti et al.^21^, 2007 | * | * | * | * | - | * | * | * |
| Paulsen et al.^22^, 2006 | * | * | * | * | * | * | - | * |
| Sorbe et al.^23^, 2004 | * | * | * | * | ** | * | * | * |
| Flynn et al.^15^, 2002 | * | * | * | * | ** | * | * | * |
| Warwick et al.^24^, 1995 | * | * | * | * | - | * | * | * |

A study could be awarded a maximum of one star for each item except for the item Control for important factor or additional factor. The definition/explanation of each column of the Newcastle-Ottawa Scale is available from (http://www.ohri.ca/programs/clinical_epidemiology/oxford.asp.).

^a^ A maximum of 2 stars could be awarded for this item. Studies that controlled for International Federation of Gynecology and Obstetrics (FIGO) stage received one star, whereas studies that controlled for other important confounders such as residual disease received an additional star.

^b^ A cohort study with a median follow-up time >24 months was assigned one star.

^c^ A cohort study with a follow-up rate >75% was assigned one star.
